# Fats and Oils as a Sustainable Source of Photopolymerizable Monomers

**DOI:** 10.3390/polym16243570

**Published:** 2024-12-20

**Authors:** Alberto Spessa, Franca Castiglione, Alessandra Vitale, Roberta Bongiovanni, Sara Dalle Vacche

**Affiliations:** 1 Department of Applied Science and Technology, Politecnico di Torino, Corso Duca degli Abruzzi 24, 10129 Torino, Italy; alberto.spessa@polito.it (A.S.); alessandra.vitale@polito.it (A.V.); roberta.bongiovanni@polito.it (R.B.); 2 Departiment of Chemistry Materials and Chemical Engineering “Giulio Natta”, Politecnico di Milano, Via Luigi Mancinelli 7, 20131 Milano, Italy; franca.castiglione@polimi.it; 3 INSTM—Politecnico di Torino Research Unit, 50121 Firenze, Italy

**Keywords:** biobased monomers, photopolymerization, photocurable monomers

## Abstract

Bio-derived monomers and biobased building blocks obtained from natural sources, e.g., fats and oils, are attracting increasing attention mainly due to sustainability concerns. Due to their features, renewable feedstocks are an excellent alternative to petroleum-based raw materials to shift towards greener chemistry, especially when coupled with energy-efficient processes like photopolymerization. In this review, we illustrate the recent research outcomes in the field of photocurable biobased monomers, showing the advantages of using biobased chemicals for the synthesis of photocurable monomers and the potential of naturally derived building blocks in photocuring reactions.

## 1. Introduction

Nowadays, polymers and polymeric networks play a pivotal role in society, being employed for several applications ranging from commodities production to the aerospace sector, improving comfort and facilitating human lives. However, since the processes have historically relied on employing oil-based raw materials and are often polluting, energy-intensive and/or potentially hazardous, they are frequently regarded as being harmful to the environment and to human health. In this scenario, the use of renewable feedstock, such as biomass-derived raw materials, for the production of green polymeric networks is thus necessary, coupled with the use of sustainable processes exploiting safer and more environmentally friendly technologies to increase energy efficiency and move towards both waste and pollution reduction [1,2].

Photoinduced polymerization processes are inherently more sustainable than thermal ones, as they are faster, can be carried out at room temperature, are solventless, and are generally more efficient [3,4]. Due to these distinctive features, over the last four decades, photopolymerization has attracted a lot of interest. The global market for photocurable resins was valued at USD 2.6 billion in 2022, and according to forecasts, it is expected to grow at a compound annual growth rate (CAGR) of 10.6% until 2030, reaching a global market share of USD 5.78 billion by the end of the decade [5]. The application range in which photopolymers can be used is constantly growing, from more common sectors like coating, printing inks, packaging, and electronics up to more innovative and challenging areas like optics and biomedical and additive manufacturing. Furthermore, the environmentally friendly features of photoinduced polymerization make it the perfect tool to employ in conjunction with renewable feedstock to produce sustainable polymers, since developing polymers from biomass is especially interesting only if it is coupled with the use of environmentally friendly processing techniques.

For photopolymerization applications, the majority of commercially accessible precursors, i.e., monomers and oligomers, are mainly produced using petrochemical raw materials, which have several disadvantages like non-renewability, increasing scarcity, and volatile and high prices. The use of bio-derived building units can lead to the development of sustainable polymers, solving problems such as the depletion of fossil fuels and reducing and reusing waste from agricultural and agri-food industry byproducts or excess production.

Fatty acids and polysaccharides are among the most developed and widely used biosource-derived monomers and polymers for the production of green polymeric materials. Several other compounds, including natural phenols and polyphenols, terpenes, and lignin and its derivatives, are also under development [6,7,8]. Plant-derived or microorganism-derived biomass, such as vegetable oils, are some examples of alternative resources for the production of precursors due to many advantages like a lower environmental impact, sustainability, and natural abundance [9]. However, bio-sourced precursors, especially the ones obtained from vegetable oils, often have poor thermomechanical properties compared to petrochemically derived ones as a result of their chemical composition, featuring long and flexible hydrocarbon chains [10,11]. The combination of them with natural fillers, especially cellulosic fibers and nanoparticles, which are lightweight, biodegradable, reasonably priced, and derived from abundant and renewable sources, is undoubtedly one of the most intriguing strategies to enhance the final properties of the polymeric network [12,13,14]. In the additive manufacturing field, UV-curable formulations based on several biobased precursors have been recently proposed [15,16,17,18,19]. Starting from various bioproducts, mainly soybean oil but also vanillin or lignin, different (meth)acrylic resins were developed for vat photopolymerization [20]. Moreover, the use of biobased monomers or oligomers can enable the design of polymeric networks with degradation features, enhancing the chances of recycling the final polymerized material and overcoming the limitations set by petrochemically derived precursors [21].

This review aims to report the current knowledge on the use of plant-derived precursors for the obtainment of biobased photocurable monomers. In the first part, the principles of photopolymerization and main photoinduced polymerization processes are reviewed. In the second part, the chemical modification of bio-sourced monomers, in particular of vegetable oils and fats, is discussed, leading to the introduction of photocurable functionalities, thus allowing for their photocuring. Photopolymerization reactions of different biobased monomers are also described.

## 2. Photoinduced Polymerization

According to the International Union of Pure and Applied Chemistry (IUPAC) glossary of terms [22], photoinduced polymerization is defined as the polymerization of a monomer by a free radical or ionic chain reaction initiated by photoexcitation, and thus from the production of a reactive species by the absorption of ultraviolet, visible, or infrared light. Thus, photoinitiators play a pivotal role in determining the extent of photoinduced polymerization reactions. Reports of quantum yields for specific systems are occasional in the literature, and the prediction of photochemical conversion is rarely performed, even when data are available. Considering exclusively the initiation process for the same photoinitiator, the initiation rate is the result of several factors, such as dissociation quantum yield, light wavelength and intensity, etc. [23,24]. Applicable predictive methods are still lacking, although photopolymer chemists are aware that accurate predictions are needed to unlock the full potential of photochemical reactions [25].

Nowadays, photoinduced polymerization is widely recognized as a green technology, owing to several environmental aspects, among them the lower energy requirements, high reaction rates, and lower volatile organic compound (VOC) emissions compared to traditional thermal polymerization processes [3,4]. Irradiation with UV light enables fast chemical reactions and molecular processes, besides bond cleavage, isomerization, and cyclization [24]. However, compared to thermally induced processes, photopolymerization is limited by the penetration depth of the light and thus its absorption by the material. Photoinduced polymerization processes are therefore commonly used to produce thin films, while 3D structures are only achieved by curing layer-by-layer [3,26]. Photopolymerization allows for convenient spatial and temporal control of the polymerization process, since reactions can be started and finished by simply switching on and off the light source. Moreover, the response is confined to areas that are illuminated by light, avoiding an uncontrolled polymerization reaction [27]. All of these features make this polymerization process suitable for applications like photolithography or 3D printing, like stereolithography (SLA), digital light processing (DLP), or direct laser writing (DLW). In photolithography, patterns are created on a photocurable polymer film by selectively illuminating it through a lithographic mask, while with 3D printing processes, three-dimensional structures are built by selectively illuminating portions of a photocurable resin in a reservoir [28,29,30,31].

Additionally, photopolymerization is a key enabling technology for those applications in which thermal-induced polymerization is not always applicable, such as dentistry, tissue engineering, or drug delivery [32]. Although UV light sources are often used for photopolymerization, there is a growing movement towards the use of visible light sources due to safety and energy-saving concerns [4,33,34].

### 2.1. Chain Growth Polymerization

**Figure 1:**
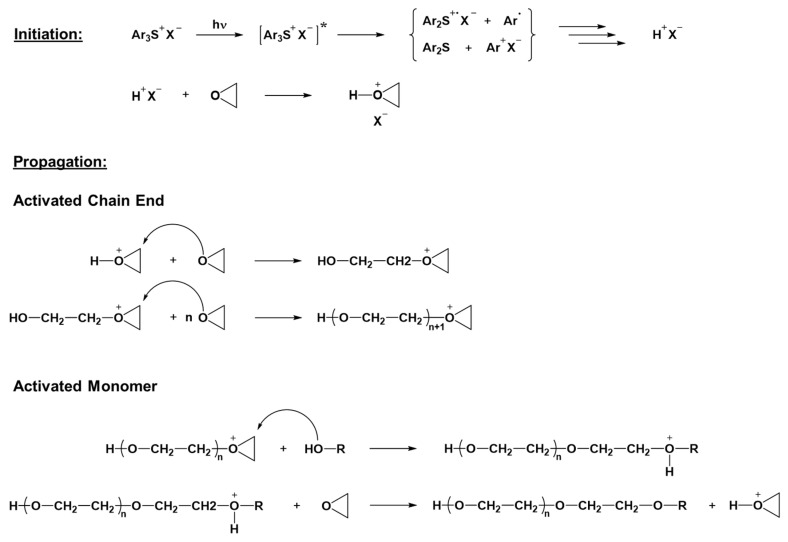
Initiation and propagation mechanisms for the cationic photopolymerization of epoxides. Adapted with permission from [40], copyright John Wiley and Sons (Hoboken, NJ, USA).

Most photoinduced polymerization is driven by chain growth processes. In this polymerization process, the reaction starts by opening up a carbon–carbon double bond or a ring structure of the monomer. The active center generated then reacts with another monomer, transferring the active center to the second monomer, which is added to the first one, creating a dimer. The reaction continues until the termination phase, and thus quenching of the active site is reached. Typically, the polymerization process can be divided into three different phases, namely the initiation, propagation, and termination. With the chain growth process, a high molecular weight can be achieved rapidly, even when the monomer conversion value is still low [35,36,37]. In the case of photoinduced chain growth polymerization, the reactive species that can start the polymerization reaction may be ions or radicals, generated upon irradiation with light of appropriate wavelengths, mainly in the UV region. Photoinduced cationic polymerization has been extensively studied since the discovery in the late 1970s, by Crivello and his co-workers, of the initiating capabilities of aryl iodonium salts towards epoxide groups when exposed to UV light [38]. Typical monomers employed for cationic photopolymerization are the ones that can be polymerized through a cationic chain growth mechanism, like epoxides, vinyl ethers, or oxetanes. Among all these classes of compounds, epoxides are the ones mostly used for practical applications. Generally, the photolysis of onium salts upon UV light exposure is followed by the hydrogen abstraction and production of a strong Brønsted acid, as reported in the simplified mechanism depicted in Figure 1 [39].

The photogenerated Brønsted acids act as the reactive species capable of starting the polymerization, which then proceeds using a chain growth mechanism. In the case of the presence of epoxide monomers, cationic photopolymerization occurs by the ring opening of the epoxide groups, following the so-called active chain end mechanism, as depicted in Figure 1 [41]. If alcohols or other compounds with hydroxyl groups are also present, the reaction follows a different route, known as the activated monomer mechanism, in which the nucleophilic attack of the alcohol on the oxirane ring produces a protonated ether. The protonated ether then transfers the proton to another epoxide ring, resulting in the termination of the growing polymer chain. This phenomenon is known as a chain transfer reaction, since the newly protonated monomer can then start a new polymeric chain reacting with other monomers (Figure 1) [40]. Cationic photocuring has several advantages; the reaction is not inhibited by oxygen, and once initiated, polymerization proceeds even without light (dark curing) due to the long-lived Brønsted acid ensuring a high conversion value. Moreover, the ring-opening reaction reduces the polymerization shrinkage, lowering the residual stresses in the final networks [42]. On the other hand, the photolysis of onium salts is more efficient at short- and mid-UV wavelengths (200–300 nm); the need for such energetic and dangerous UV radiations constitutes the main disadvantage of cationic photopolymerization [43].

The development of photoinitiating systems with absorbance ranges shifted in the visible region has been found as a solution to overcome these issues. Two mechanisms can be exploited, namely the introduction of photosensitizers and the use of free radical-promoted cationic photopolymerization (FRPCP) [34,43,44,45,46]. In the first strategy, photosensitizer molecules, like polycyclic hydrocarbons, highly conjugated heterocyclic compounds, and dyes, are added into the photocurable system to activate the cationic photoinitiator by the electron transfer between the excited photosensitizer and the onium salts [43]. Instead, FRPCP involves free radical generation by the photodegradation of a radical photoinitiator. Once radicals are produced, the cationic polymerization is started either by an addition–fragmentation mechanism with the addition of radicals to an allylic onium salt and the subsequent fragmentation of it or by an electron transfer mechanism, in which the onium salt oxidizes the free radical [45,47].

**Figure 2:**
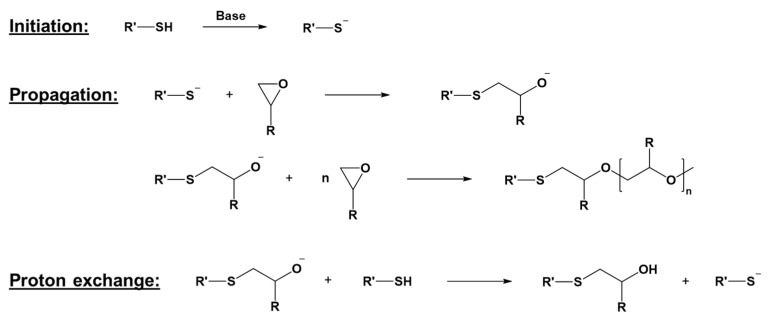
Thiol-epoxy anionic photopolymerization mechanism. Adapted under the terms of the CC−BY license from [48].

Besides cationic photopolymerization, anionic chain growth reactions are a promising chemistry with peculiar advantages. An example of this kind of mechanism can be found in the thiol-epoxy ring-opening reaction (Figure 2). However, practical applications of anionic photopolymerization have not yet been developed, mainly due to the lack of suitable photoinitiators.

**Figure 3:**
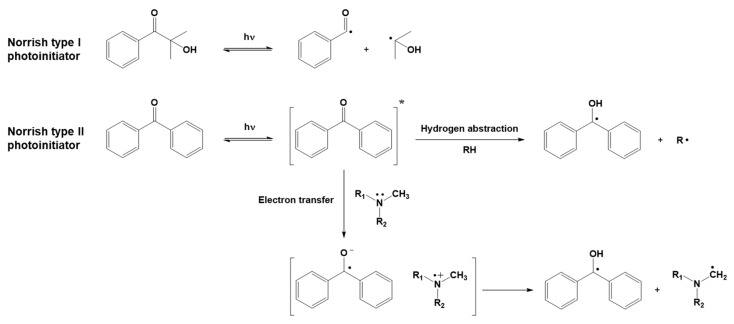
Generation of radicals through the photolysis of Norrish type I and Norrish type II photoinitiators.

Free radical photoinduced polymerization is arguably the most widely used photoinduced polymerization reaction mechanism. The monomers which photopolymerize via a free radical mechanism are those containing at least one carbon–carbon double bond in the chain, such as (meth)acrylic monomers or unsaturated polyesters. The dissociation of a light-reactive molecule, i.e., the photoinitiator (PI), generates the radical initiating species, as shown in Figure 3. Depending on the mechanism of radical generation, radical photoinitiators are divided into two classes [49], called Norrish type I and Norrish type II.

For Norrish type I photoinitiators, radicals are generated by a unimolecular process: the homolytic cleavage of the photoinitiator molecule produces two radical species. Norrish type II photoinitiators, on the other hand, produce radicals by a bimolecular process: after activation by light, radicals are generated by a photoinitiator molecule reacting with a co-initiator via processes such as hydrogen abstraction or electron transfer.

**Figure 4:**
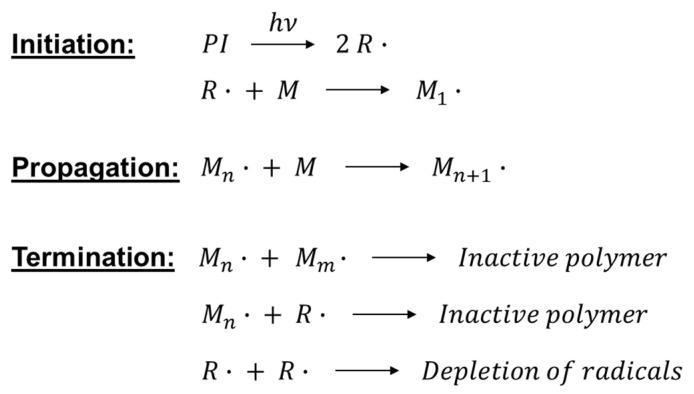
Free radical photopolymerization mechanism.

The initiating radicals react with the monomer generating the propagating species; the chain growth mechanism is sustained until the rate of the termination reaction exceeds the rate of propagation, leading to the end of the polymerization (Figure 4).

The free radical mechanism allows both spatial and temporal control of the polymerization reaction, since the radicals are short-lived and their concentration decays rapidly in the absence of light. Under a non-inert atmosphere, radicals can be quenched by oxygen; this leads to the presence of a less cured surface layer when the polymerization reaction is carried out in air. This, which is one of the major disadvantages of radical photopolymerization, was turned into a valuable tool to obtain multi-polymeric patterns and devices by photolithography [50]. Other drawbacks of this mechanism are significant polymerization shrinkage and the presence of unreacted monomers or photoproducts produced during the cleavage of the photoinitiator [3].

### 2.2. Thiol-Ene Click Polymerization

**Figure 5:**
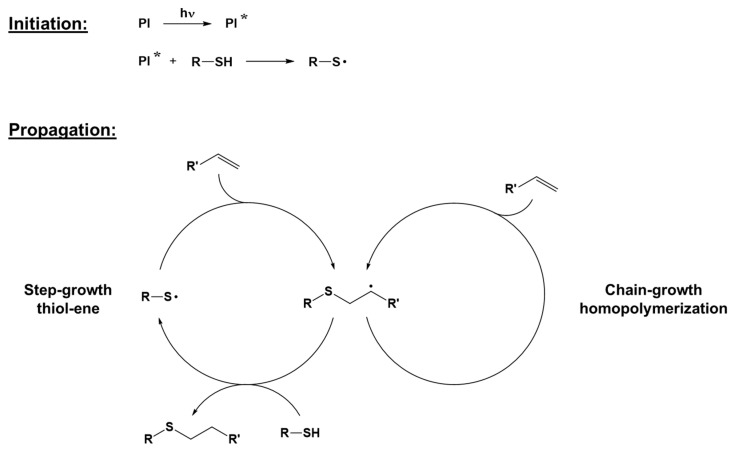
The general mechanism of radical thiol-ene click polymerization. Adapted with permission from [55,56], copyright Elsevier (Amsterdam, The Netherlands) and from [57,58], copyright John Wiley and Sons (Hoboken, NJ, USA).

The thiol-ene click reaction is a type of polymerization in which the crosslinking reaction occurs between a stoichiometric mixture of a multifunctional thiol-containing molecule and a multifunctional olefin, alternating propagation and chain transfer steps during the reaction [51]. The thiol-ene photoinduced reaction may also be performed in off-stoichiometric conditions, working in excess of either thiol or ene, enabling unreacted functionalities that can be used for post-polymerization and further surface functionalization [52]. Although the thiol-ene reaction can occur through different reaction mechanisms, e.g., thiol-Michael reactions or radical mechanisms, this review focuses on the more common radical process. Photoinitiators typically used for photoinduced thiol-ene polymerization are of the abstraction type, since hydrogen abstraction and thus thiyl radical formation, are necessary to start the reaction [53]. Benzophenone is one of the most used photoinitiators: after the excitation of benzophenone due to light exposure, it abstracts a hydrogen atom from thiol, forming a thiyl radical able to add to the ene structure and start the polymerization reaction, regenerating another radical. Disulfides can also be employed as photoinitiators due to their ability to form two active thiyl radicals upon photoexcitation [54,55]. In the propagation step, thiyl radicals are added to the alkene, forming a thioether. The newly formed carbon radical can then either abstract hydrogen from a thiol and regenerate a thiyl radical or attack another alkene, propagating the radical through a free radical reaction mechanism [55]. While the latter can be identified as a chain growth mechanism, the former reaction is defined as a step-growth reaction. A general overview of the process is reported in Figure 5. Unlike the chain growth process, with step-growth polymerization, molar weight increases only at the later stages of polymerization [35,56].

The presence of electron-withdrawing groups near the double bond of enes (e.g., acrylates or methacrylates) facilitates the chain growth homopolymerization, while electron-donating substituents or strained bonds (e.g., vinyl ethers or norbornene) favor a step process [55]. Therefore, the final properties of the cured material can be finely tuned by a careful selection of both thiols and enes involved in the reaction.

Unlike free-radical polymerization, thiol-ene is not inhibited by the presence of oxygen, overcoming the necessity to work in an inert atmosphere, reaching a higher level of crosslinking. The final photocured polymeric network is homogeneous, with reduced shrinkage and internal stresses, leading to a narrower glass transition region [52,59,60]. Furthermore, by properly selecting the wavelength and the monomer type, a photoinitiator-free self-initiating formulation can be prepared, allowing for the production of optically clear and transparent films [54,59].

### 2.3. Photocycloaddition

Cycloadditions are reactions in which independent π systems combine, leading to the formation of a cyclic ring, reducing the bond multiplicity [61]. Cycloadditions can be obtained either thermally or photochemically, depending on the nature of the molecule involved in the reaction. Among thermally allowed cycloadditions, [4+2] Diels-Alder, combining a diene and a dienophile, is the most used one.

The coupling and subsequent ring formation can be activated in specific molecules upon light exposure. In this specific case, the process is called photocycloaddition. Some naturally occurring compounds go through a photoinduced reversible [2+2] cycloaddition reaction, e.g., coumarins and cinnamates. Stereochemistry plays an important role in photocycloadditions. As an example, both *cis* and *trans* isomers of cinnamic acids exist, of which *trans-*cinnamic acid is more common in nature and more stable [62]. Dimerization through photocycloaddition is favored for the *trans* isomer, while for the *cis* isomer, geometrical isomerization is more commonly observed, competing with dimerization [63].

**Figure 6:**
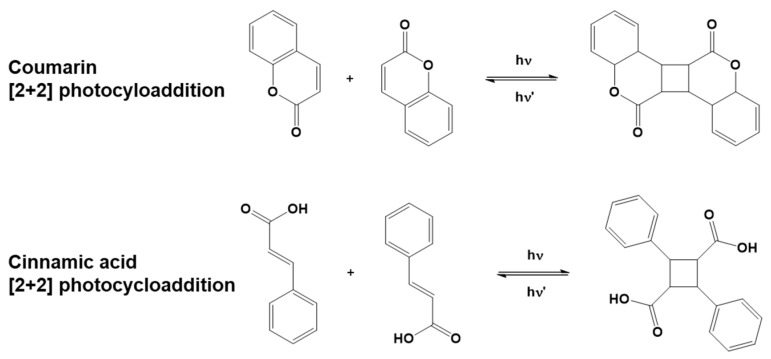
Examples of coumarin and cinnamic acid [2+2] photo-reversible cycloaddition reactions.

Besides the photoinduced reactions listed so far, photocycloaddition is a different way to obtain a crosslinked polymeric structure, exploiting the ring formation (Figure 6) as an alternative way to induce crosslinking between linear polymeric chains (i.e., thermoplastic polymers). The photocrosslinking of linear polymers by cycloaddition can also involve reversible bonding, allowing for the disassembly of the networks formed [64].

Derivatives of coumarins and cinnamates have been employed to functionalize thermoplastic polymers, enabling photocrosslinking. This method was recently extended to biobased polymers, following its initial development for oil-based polymers.

## 3. Fats, Oils, and Other Lipids as Sources for Photocurable Monomers

Fats and oils from vegetable or animal sources are currently the primary renewable raw materials for the synthesis of UV-curable precursors, mainly due to their abundance and affordability [15]. Among fats and oils, a big distinction must be made between edible and non-edible compounds. While edible oils are extracted and used in the food industry, non-edible ones are mostly unexploited, and their applications are highly limited. In this scenario, the use of oil from non-edible sources as a raw material for the production of photocurable monomers may be a better alternative to avoid competition with food resources production, decreasing environmental issues like soil consumption and exploitation. In particular, wastelands can be allocated to the cultivation of non-edible crops, contrasting deforestation phenomena and avoiding direct competition with food production processes [15,65].

### 3.1. Natural Sources

Lipids can be divided into two different categories based on their chemical composition and the number of products generated by their hydrolysis. Simple lipids, composed of carbon, hydrogen, and oxygen, can lead to the generation of two products upon hydrolysis. Triglycerides, waxes, sterols, and terpenes are part of this class. Complex lipids containing additional phosphorous or nitrogen in their molecular structure can be hydrolyzed into three or more products. Phospholipids and glycolipids are two examples of complex lipids.

**Figure 7:**
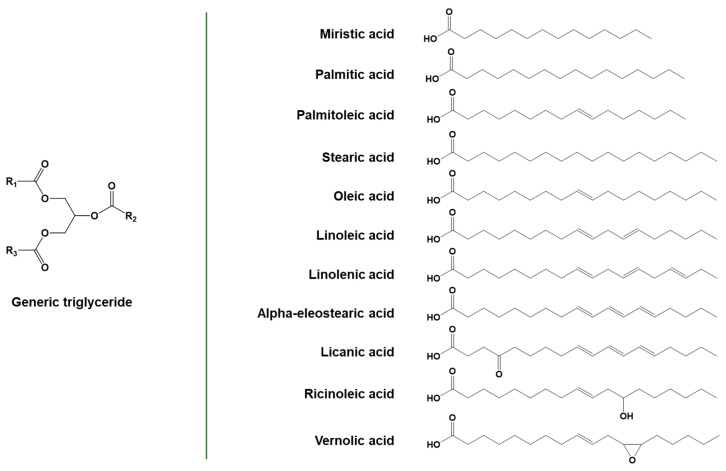
General structure of triglycerides and some common fatty acids. Adapted under the terms of the CC−BY license from [67].

The majority of vegetable oils are composed of triglycerides, a class of chemical compounds having a three-armed star structure constituted by fatty acid chains linked together by a glycerol unit through ester linkages. These covalent bonds can be hydrolyzed with simple chemical procedures, leading to the formation of glycerol and fatty acids. The latter constitute about 95% of the total mass of triglycerides. Depending on the source of the triglycerides, the aliphatic chain of fatty acids can have different lengths and degrees of unsaturation, with the number of carbon atoms typically ranging from 14 to 18 and the number of double bonds per chain between 0 and 3. Oleic, linoleic, and linolenic are some of the most commonly occurring unsaturated C18 fatty acids, containing, respectively, one, two, or three unsaturations in the chain [66]. Examples of common fatty acids are reported in Figure 7.

In vegetable oils, unsaturations are primarily present in the cis configuration, even if the trans configuration is also possible, as in the case of α-eleostearic acid or punicic acid. The direct polymerization of fatty acids exploiting their unsaturations can be complex for different reasons, such as the presence of allyl hydrogens, which act as radical traps, decreasing the reactivity of the molecules, and the huge steric hindrance of the aliphatic chain. Regarding the reactivity of the carbon–carbon double bond, the internal unsaturations have lower reactivity than the terminal ones for polymerization reactions. Still, they can be exploited for obtaining chemical modification of the fatty acid chain [6]. Besides unsaturations, some fatty acids contain other functional groups, such as epoxides (e.g., vernolic acid in vernonia oil) or hydroxyls (e.g., ricinoleic acid in castor oil), which can be useful for further functionalization or chemical modification [15,68]. The functionalization of two abundantly produced edible oils, soybean oil and linseed oil, is already at the commercial scale. Well-known examples of non-edible vegetable oils are jatropha oil, castor oil, and moringa oil [69]. Their chemical modification to obtain monomers is being extensively researched but has not yet reached the industrial scale.

**Figure 8:**
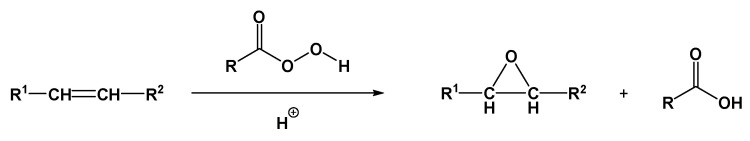
Example of an epoxidation reaction of an unsaturation using peroxides. Adapted under the terms of the CC−BY license from [70].

Among the most common derivatives of triglycerides are epoxides obtained by oxidizing the double bonds using hydrogen peroxide or peracids (Figure 8).

**Figure 9:**
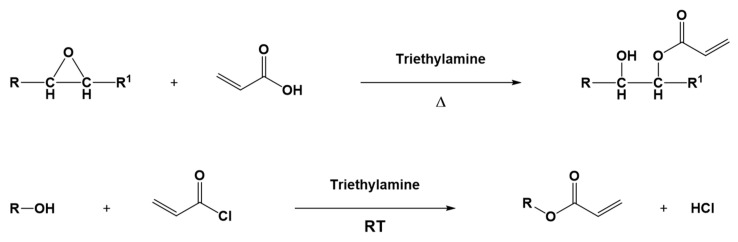
Example of acrylation reactions on epoxy group (top) or hydroxyl group (bottom). Adapted under the terms of the CC−BY license from [74] and with permission from [75], copyright John Wiley and Sons (Hoboken, NJ, USA).

Epoxidized vegetable oils are also used as an intermediate point for the synthesis of other biobased photocurable monomers. Indeed, (meth)acrylated derivatives of triglycerides are generally obtained from the corresponding epoxidized ones by a straightforward reaction with acrylic or methacrylic acid, as reported in Figure 9 [71]. Also, hydroxylated oils, e.g., castor oil, can be transformed into the corresponding acrylic molecule, in this case performing an esterification reaction through the use of acrylic acid or acryloyl chloride (Figure 9) [72,73].

Terpenes, terpenoids, and rosins are a wider class of unsaturated organic compounds typically found in many essential oils, such as limonene, pinene, myrcene, or linalool. They can be extracted from both edible and non-edible sources; as an example, limonene can be extracted from the peel of citrus fruits, while α-pinene from non-edible turpentine oil [76]. The chemical structure of terpenes is constituted by the repetition of different isoprene units connected in linear or cyclic structures [77,78]. Their structural diversity and thus the different reactivity of carbon–carbon double bonds make them extremely attractive and versatile for different functionalizations. In this scenario, terpenes are some interesting candidates for use in polymerization reactions [79,80]. The synthetic functionalization of terpene structures provides another possibility for the production of photopolymerizable monomers [81]. As previously discussed for vegetable oil unsaturation, double bonds present on the terpene structure can be used directly in thiol-ene click polymerization or they can be further functionalized, e.g., by introducing some epoxy moieties. The oxidation of d-limonene with the consequent production of limonene oxide is just an example of the introduction of epoxide functionalities on terpene structures [21].

Among natural compounds, the potential of natural phenolic compounds to replace oil-based monomers such as phenol and its derivatives, which are frequently employed in the production of phenolic resin, has been investigated. Natural phenols can be extracted from biomass generated in the agricultural sector (e.g., cashew nutshells, palm trash, coconut shells) and the paper industry (lignin). Examples of such compounds include cardanol, eugenol, vanillin, and resorcinol [82].

Cashew nutshell liquid (CNSL), extracted from nutshells, a non-edible byproduct of the cashew industry, is one of the most abundant sources of natural phenolic compounds, accounting for approximately 20% of the total mass of the cashew nut [83], with an annual output of over 10 million tons [84]. One of the primary components of CNSL is cardanol, an alkylphenolic chemical which can be recovered by heat treatment. From a chemical point of view, cardanol is a combination of four meta-alkylphenols with varying degrees of aliphatic chain unsaturation, primarily in the cis conformation. Cardanol’s distinctive structural composition, comprising an aromatic head (phenol) and a C15 aliphatic chain with unsaturations, enables functionalization at both the phenolic hydroxyl and the fatty chain double bonds. The epoxidation of cardanol can be performed in different ways, exploiting the various reactive functionalities present on the cardanol molecule. Peroxides can facilitate the conversion of unsaturations to epoxide groups [85,86]; however, these epoxy functionalities situated within the aliphatic chain are not particularly reactive. In contrast, the phenolic hydroxy functionality of cardanol can be epoxidized via a direct reaction with epichlorohydrin [87,88] to produce a more reactive phenolic glycidyl ether. Some monomers and oligomers containing only those functionalities are commercially available [89,90].

Eugenol and its derivatives are another class of compounds which have gained importance for the obtainment of biobased monomers. Eugenol is a phenolic compound that can be obtained through extraction from several plants (i.e., cloves, nutmeg, cinnamon, pepper, basil, marjoram, etc.) or depolymerization from lignin [75,91,92,93]. Eugenol is composed of a single molecule, which includes an aromatic ring with three different substituents, namely a phenolic hydroxyl, a methoxy group, and an alkyl chain with an allylic double bond [94]. The inherent allyl group can be particularly useful for further functionalization of the eugenol molecule, like epoxidation or a thiol-ene click reaction. Moreover, the phenolic group can also be used to introduce a glycidyl ether group [94]. Its ability to be functionalized with one or more epoxy groups makes it a promising candidate for the replacement of bisphenol A diglycidyl ether (DGEBA) as an epoxy resin [95]. The thiol-ene click reaction was used to prepare eugenol methacrylic derivatives by first reacting the allylic double bond with carboxylic acids containing a thiol functional group and then acrylating with glycidyl methacrylate [94]. (Meth)acrylate functionalities can also be introduced on eugenol and eugenol derivatives by hydroxyethylation of the phenolic hydroxyl with ethylene carbonate and a subsequent reaction with methacrylic anhydride [75]. The additional advantage of this technique relies on the avoidance of the radical scavenging activity of the methacrylic anhydride [96,97]. The synthesis of eugenol-based dimers, trimers, or multiarmed structures bearing multiple epoxy and allylic groups was reported in previous works [85,98,99].

Another phenolic chemical compound which is commonly extracted from biomass is vanillin. While vanillin can be extracted from vanilla beans, this production is very limited. Lignin has recently emerged as a source of biobased vanillin, and wood-based vanillin is already commercially available [100]. Vanillin-derived diglycidyl ethers can be easily obtained by exploiting hydroxyl functionalities present on either the vanillin structure or on vanillin alcohol derivatives obtained through reduction/oxidation reactions [101,102]. Further functionalization of alcohol derivatives can lead to the production of allylated compounds, which are particularly useful for thiol-ene or radical polymerization. The methacrylation of vanillin using methacrylic anhydride was also reported [103,104].

### 3.2. Recycled Sources

Recently, it has been found that used vegetable oils (UVOs) are a valuable source of long-chain unsaturated fatty acids (mainly linoleic, linolenic, and oleic) in the form of tri-/di- and mono-glycerides and a variable percentage of saturated fatty acids (SFAs). UVOs, also known as waste cooking oils (WCOs), are a mixture of different kinds of exhausted vegetable oils and are by-products of the food chain, which arise from private kitchens and catering industries from deep frying cooking processes. Changes in the chemical composition of vegetable oils during the frying process may be related to food contamination and decomposition reactions. However, the relative amount of impurities generated during the cooking process is not high and can be removed.

**Figure 10:**
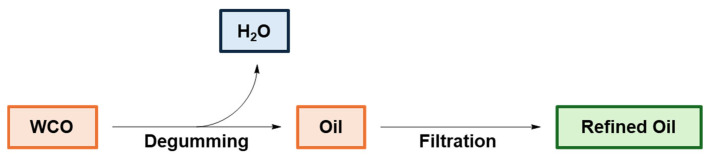
Process for WCO purification. Adapted under the terms of the CC−BY license from [105].

In view of recovering fatty acids and triglycerides from WCOs, purification is a required key step. The entire process, illustrated in Figure 10, includes the water extraction of chemicals from the crude WCO (degumming) and/or filtering with cellulose or bentonite to decrease the oil density and viscosity by removing selected polar compounds [105,106]. Furthermore, the chemical composition and physico-chemical properties of the recycled oil are sensitive to experimental degumming conditions, such as water pH and temperature [107]. The refined oil is then characterized by gas chromatography and proton high-resolution nuclear magnetic resonance (^1^H NMR) spectroscopy [108].

In Table 1, the fatty acid composition of sunflower commercial oil (SCO) and purified WCOs (with different pH conditions), determined by ^1^H NMR spectroscopy, is reported. The chemical composition of WCOs is quite similar to that of the edible oil used as a reference, as a significant amount of unsaturated fatty acids has been preserved despite the frying process. However, relevant changes in relative concentration are observed for linoleic acid, whose concentration decreases, and oleic acid, for which an increase is observed.

## 4. Synthesis of Photocurable Monomers from Natural and Recycled Sources and Their Polymerization

### 4.1. Vegetable Oils

Epoxidized vegetable oils (EVOs) are employed as environmentally friendly plasticizers, stabilizers, and lubricants [109,110], as well as sustainable biobased alternatives to fossil-based epoxy resins [66]. Due to their aliphatic nature, EVOs possess flexible structures and relatively low reactivity, as the epoxide groups are located on the aliphatic chain. Consequently, the materials obtained by their homopolymerization typically display low mechanical properties, and EVOs are mainly mixed with other co-monomers or used as reactive diluents in photocurable formulations. Moreover, due to their weak mechanical properties, they are commonly employed for non-structural applications such as coatings, inks, and adhesives [68,111]. In particular, epoxidized soybean oil (ESO) and epoxidized linseed oil (ELO) are now the most significant products on an industrial scale [8,66]. Epoxidized triglyceride oils have been cured by cationic UV light since the early 1990s, when their photocuring was reported for the production of flexible, transparent thin films [112]. Chemically modified oils, as well as naturally occurring epoxidized oils, such as soybean, linseed, and vernonia oils, were included in the study [112,113]. Afterwards, vegetable oils and the corresponding epoxide derivatives were extensively studied as monomers and oligomers for cationic photopolymerization; soybean, linseed, castor, safflower, sunflower, canola, lesquerella, vernonia, and rapeseed oils are just some examples [112,114,115,116]. Among them, epoxidized castor oil (ECO) exhibited excellent reactivity. Indeed, the hydroxyl groups present on the fatty acid chains of ECO allow the photopolymerization to proceed by the activated monomer mechanism [114]. Photocured ECO resulted in a flexible network, having a glass transition temperature slightly lower than room temperature, limiting vitrification phenomena during curing and resulting in a value of the conversion of epoxy groups at around 85% [117]. To foster the exploitation and then the fields of application of biobased UV-cured coatings, epoxidized rosehip seed oils and epoxidized grape seed oils were used for the production of photocurable coatings with anti-corrosion properties. When higher crosslinking densities were reached, besides good corrosion resistance, these UV-cured coatings showed high solvent resistance, surface hardness, and an effective adhesion to the metallic substrate [118]. Different EVOs, such as epoxidized linseed oil, epoxidized corn oil, and epoxidized soybean oil, were used as monomers for stereolithography 3D printing using visible light, exploiting the FRPCP mechanism by coupling a visible light photoinitiator and a mixture of an onium salt and tris(trimethylsilyl)silane as cation generators [119].

A different type of photoinduced reaction involving thiol groups is thiol-epoxy, an anionic epoxide ring-opening polymerization which has been recently used for the production of polymeric networks exploiting an anionic photoinitiated mechanism. The thiol-epoxy reaction of epoxidized linseed oil with different thiols, like 1,3-benzenedithiol and pentaerythritol tetra(3-mercaptopropionate), enabled a high-resolution 3D printing of components with laser direct writing, having individual features with a 100 nm resolution [120]. Another example of this chemistry is the production of photocurable coatings through the reaction between epoxidized cottonseed oil and 7-mercapto-4-methyl coumarin, which showed a self-healing ability mainly due to coumarin [2+2] cycloaddition [121].

Epoxidized soybean oil was reacted with *trans*-cinnamic acid, obtaining a grafting ratio of 2.61 cinnamic moieties per molecule. Exploiting the photocycloaddition reaction of *trans*-cinnamic acid, a flexible coating for PET was produced showing good adhesion and UV-shielding performance [122].

Acrylated and methacrylated compounds are suitable for photoinduced free radical and thiol-ene polymerization reactions. Recently, (meth)acrylated epoxidized soybean oils have been proposed as a biobased alternative for lithography-based 3D printing processes, such as laser-based stereolithography (SLA) and digital light processing (DLP). Recent studies have reported the use of these soybean oil-derived monomers in 3D printable formulations. These monomers have been mixed in different ratios with standard petroleum-based resin to improve the environmental profile [20,123]. Alternatively, they have been combined with biobased reactive diluents, e.g., acrylated eugenol [124] or tetrahydro furfuryl methacrylate [125], to obtain thermomechanical performances comparable to commercial SLA resin. Furthermore, AESO resin was used for the production of shape memory biocompatible scaffolds through a laboratory 3D laser printing apparatus [126]. Besides 3D printing and vat photopolymerization, acrylated vegetable oils such as acrylated linseed oil, acrylated rapeseed oil, and acrylated grapeseed oil found applications in the wood coating sector [127,128].

In the composites field, studies on the photocuring of materials using an acrylated epoxidized soybean oil matrix reinforced with fiber and varying cellulose-to-lignin ratios revealed that photopolymerization was more inhibited by a greater proportion of lignin, which is an absorber of UV light [129]. Nevertheless, as evidenced by a recent study [130], free radical photoinduced reactions were observed to occur with remarkable ease in the presence of nanocellulose. However, it is noteworthy that the majority of the investigated matrices were derived from fossil-based sources. The reinforcement of acrylated epoxidized soybean oil with cellulose nanocrystals (CNCs) for coatings production was proposed. There was no discernible difference in the curing behavior of AESO reinforced with CNCs and unreinforced AESO. The degree of optical clarity exhibited by the material declined as the glass transition temperature increased [131,132]. When different types of cellulose reinforcement were compared, CNCs were more evenly distributed throughout AESO than microfibrillated cellulose (MFC) and exhibited a stronger mechanical reinforcing effect [133]. MFC was successfully employed for the photocuring of a preform impregnated with AESO, resulting in the production of self-standing, flexible, and moderately transparent films with good thermal stability [134]. For vinyl monomers derived from palm oil, methacrylated eugenol and micro-sized bamboo fibers were successfully mixed, forming a 3D printable photocurable resin with a glass transition temperature (T_g_) of around 100 °C. Furthermore, the photocured matrix could be successfully degraded with a simple treatment with an alkaline aqueous solution to recycle the fibers from the composite [135].

As described before, thiol-ene photopolymerization takes place between a thiol group and an unsaturated C-C bond, involving functional groups like allyls, (meth)acrylates, and vinyl ethers. Unsaturations present on the fatty acid chains of vegetable oils can be directly exploited for thiol-ene crosslinking reactions, even without further functionalization of the oil molecules [121,136]. The isomerization of unsaturations between the *cis*-*trans* isomers and vice versa has been proven to occur upon irradiation in vegetable oils, such as linseed oil [137], and can compete with the thiol-ene photopolymerization reaction. Thanks to thiol-ene click chemistry, coatings were produced by reacting internal double bonds of fatty esters with multifunctional thiol crosslinkers [137]. Thiol-ene ultraviolet (UV) curable systems with 40% biobased content were obtained by synthesizing a series of castor oil derivatives by reacting the hydroxyl groups of the fatty chain with a specific isocyanate bearing the selected vinyl group. Acrylated castor oil was cured using trimethylolpropane tris(3-mercaptopropionate), producing a flexible coating with high hardness and good solvent resistance [138]. The photopolymerization of acrylated linseed oil through a thiol click reaction with difunctional or trifunctional thiol crosslinkers for DLP 3D printing applications was also reported [139]. The esterification of the hydroxyl group of castor oil by 3-mercaptopropionic acid was reported, leading to the obtainment of castor oil-based thiol. This monomer was then used for a self-initiated photoinduced reaction, in which castor oil-based thiol acted both as a thiol and vinyl monomer during the click reaction [140]. A similar procedure was applied to synthesize a thiol from isosorbide that was polymerized with tung oil in the presence of a cationic photoinitiator [141]. Recently, allylated linseed oil was employed by Sölle and coworkers as a biobased monomer for UV-induced nanoimprint lithography (NIL), using a thiolated eugenol derivative as a crosslinker for the thiol-ene polymerization reaction [142].

### 4.2. Terpenes

**Figure 11:**
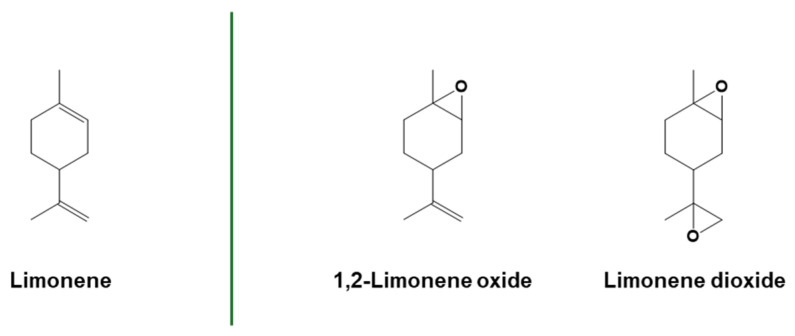
Structure of limonene and epoxidized limonene derivatives. Adapted with permission from [145], copyright Springer Nature (London, UK), and [143,144], copyright American Chemical Society (Washington, DC, USA).

One of the first terpenes used for cationic photopolymerization was limonene (Figure 11) [45]. Cationic photopolymerization was used to prepare antibacterial tack-free coatings from mono- and di-epoxy-limonene monomers, adding eugenol to the polymer network via a thiol-ene click reaction. The photocuring was started under visible light using β-carotene as a natural photosensitizer, combined with an iodonium salt photoinitiator [143]. Limonene dioxide (Figure 11) can also be photopolymerized under sunlight in ambient conditions using FRPCP mechanisms in the presence of a radical source, a silane, and an iodonium salt photoinitiator [144].

Recently, a soft, gum-like malleable polymer with a T_g_ of approximately 10 °C and good thermal stability was produced in an attempt to directly obtain poly(limonene) through the radical photopolymerization of the alkyl chain double bond using a combination of Norrish type II radical photoinitiators and alkyl halide atom transfer radical polymerization (ATRP) initiators [145].

The photoactivated thiol-ene crosslinking of terpene compounds is an example of the use of terpenes for the production of polymeric networks. In this case, multifunctional thiols are used for a thiol-ene reaction by exploiting the intrinsic double bonds present in terpenes’ chemical structure without the need for further chemical functionalization of these natural building units. [146].

### 4.3. Phenols

**Figure 12:**
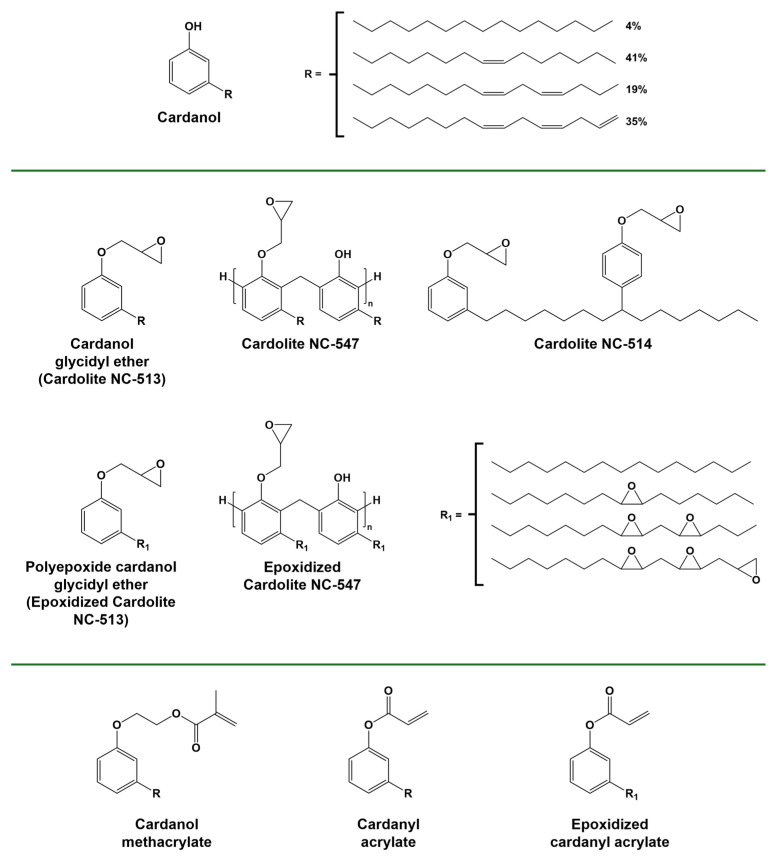
Structure of cardanol and cardanol epoxidized and/or (meth)acrylated derivatives. Adapted with permission from [88], copyright The Society of Fiber Science and Technology (Tokyo, Japan); [148], copyright Springer Nature (London, UK); [84,149], copyright Elsevier (Amsterdam, The Netherlands); and [86], copyright Royal Society of Chemistry (London, UK). Adapted under the terms of the CC−BY license from [150,151].

Cationic UV curing of commercially available cardanol-derived epoxy monomers and oligomers bearing only glycidyl ether functionality (Figure 12) has been demonstrated. Due to the long flexible chain and low functionality, the final properties of the cured coatings were relatively poor, and they were instead proposed as a co-monomer in resin formulations [147].

Cardanol and cardanol derivatives (Figure 12) epoxidized on both the phenolic site and the alkyl chain were synthesized and photocured, leading to the production of networks with relatively high conversions and insoluble fractions, having a value of T_g_ between 20 °C and 53 °C [87,88,148,152]. In the composite field, the use of epoxidized cardanol for the impregnation of commercial wood-based microfibrillated cellulose and nanocellulose preforms was reported. Although high conversion could be obtained through a cationic photopolymerization reaction, this could only be achieved in the presence of an unusually high concentration of photoinitiators. The reason behind this was attributed to a competition reaction between cellulose hydrolysis occurring due to the presence of the Brønsted acid and polymerization initiation [151,153].

Epoxidized eugenols were obtained by an epoxidation reaction of either the allylic double bond or the phenolic function using 3-chloroperbenzoic acid and epichlorohydrin, respectively. In the first case, the epoxidized eugenol was cationically photocured with a resorcinol-derived diglycidyl ether as a co-monomer to produce materials with antibacterial and antioxidant properties [154]. Eugenol diglycidyl ether, obtained by the reaction with epichlorohydrin, was also photocured using the same co-monomer, and the remaining allylic functionalities were exploited for grafting an antibacterial agent through a photoinduced thiol-ene reaction [155].

**Figure 13:**
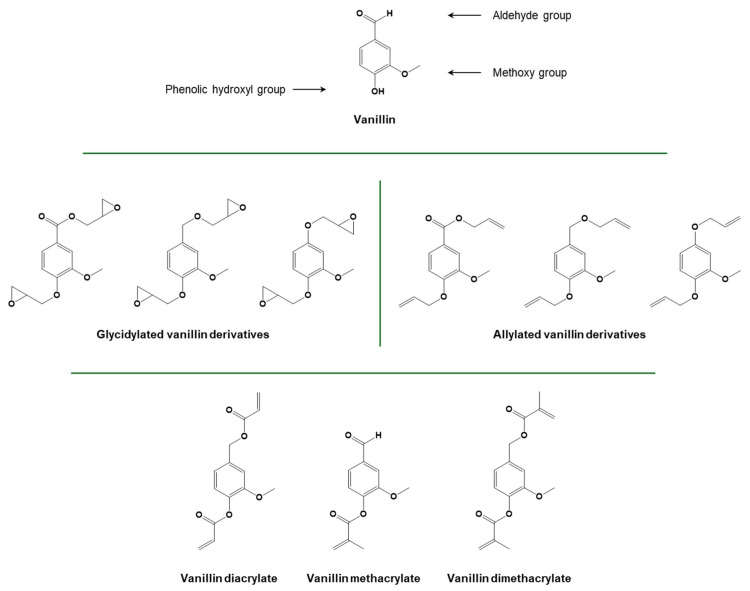
Structure of vanillin and glycidylated, allylated, and (meth)acrylated derivatives. Adapted with permission from [157], copyright Royal Society of Chemistry (London, UK), and [158], copyright Express Polymer Letters (Budapest, Hungary). Adapted under the terms of the CC−BY license from [159,160].

Additionally, vanillin alcohol diglycidyl ethers (Figure 13) have been explored as a potential substitute for epoxy resins based on bisphenol A due to their structural similarities. These monomers have recently been employed in cationic photopolymerization for the construction of photocurable networks, which exhibit glass transition temperatures higher than 50 °C [117,154,155,156].

Cardanol methacrylate (Figure 12) obtained from cardanol hydroxyethylation with ethylene carbonate and a subsequent reaction with methacrylic anhydride [161] was used for the production of clear coatings upon radical photopolymerization, although the final mechanical properties were low [150]. When the same molecule was used as an additive, it was found to increase the hydrophobicity of the UV-curable formulation [162]. Acrylated cardanol diphenyl phosphate was studied as an additive to enhance the flame-retardant properties and reduce volume shrinkage when added to urethane acrylate UV-curable coatings [148,163]. Cardanol-based acrylates and their epoxidized counterparts have been employed as reactive diluents in acrylate resins derived from vegetable oil [149,164,165]. The synthesis of cardanol-based multi-arm acrylates, in which the acrylate groups are attached to the alkyl side chain of cardanol moieties that are linked to a central core through their phenolic function, represents an intriguing method to increase the functionality and, consequently, the mechanical properties of the resulting polymer. The resulting UV-cured polymer network displays enhanced properties [166,167,168,169,170].

**Figure 14:**
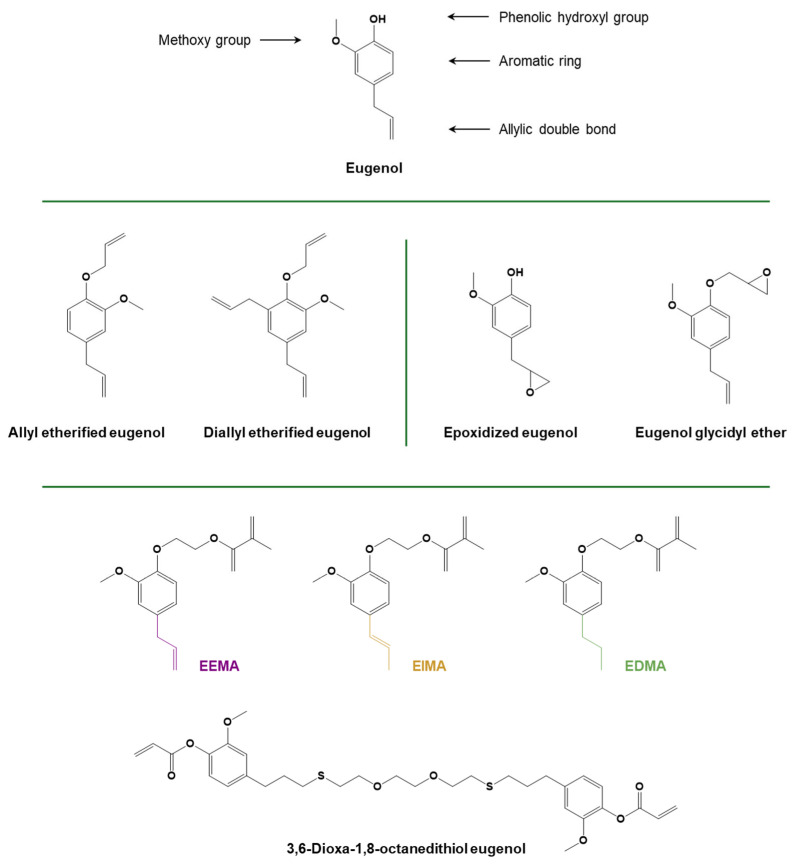
Structure of eugenol and eugenol derivatives. Adapted with permission from [154,174], copyright Elsevier (Amsterdam, The Netherlands); [94,155], copyright American Chemical Society (Washington, DC, USA); and [157], copyright Royal Society of Chemistry (London, UK). Adapted under the terms of the CC−BY license from [171].

Eugenol (meth)acrylic derivatives (Figure 14) have been widely used for free radical photopolymerization. A study was conducted to investigate the radical photocuring of methacrylated monomers derived from eugenol derivatives, namely ethoxy eugenyl methacrylate (EEMA), ethoxy isoeugenyl methacrylate (EIMA), and ethoxy dyhydroeugenyl methacrylate (EDMA). The work highlighted the role of the unsaturation on the side chain and its network position when acrylated eugenol derivatives were reacted under an inert atmosphere or air, with and without a photoinitiator [171]. Recently, the use of eugenol and vanillin-derived low-molecular-weight monomers, i.e., eugenyl methacrylate and vanillyl alcohol methacrylate, was reported for the production of a hydrophobic coating for paper and packaging applications [172]. Moreover, Dai and coworkers demonstrated that an enhancement in the thermomechanical properties of the photocured AESO-based coatings could be reached by adding eugenol methacrylic derivatives as co-monomers in the UV-curable formulation [173]. A eugenol-based diacrylate was synthesized by Ding and coworkers [157] by dimerizing eugenol with a dithiol compound, with the aim of designing a monomer with physical properties comparable with those of bisphenol A-based acrylates. A sustainable fast-curing formulation for SLA 3D printing was then prepared using this monomer, together with guaiacol methacrylate acting as a mono-methacrylate diluent and vanillyl alcohol dimethacrylate or trimethylolpropane trimethacrylate as crosslinkers, as well as a radical photoinitiator [157].

Several eugenol derivatives (Figure 14) were used to create a polymeric network through a photoinduced thiol-ene reaction, namely allyl-etherified eugenol derivatives [174], a hexa-eugenol-substituted cyclophosphazene monomer [99], and a trifunctional allyl compound synthesized by reacting eugenol with phosphorus oxychloride (POCl_3_) in an aqueous sodium hydroxide solution [175].

In the context of radical and thiol-ene photopolymerization, vanillin derivatives in the forms of acrylate and methacrylate have been employed [120,158,159,160]. Vanillin diacrylate was found to possess characteristics analogous to those of commercial petroleum-derived polymers employed in 3D printing, rendering it suitable for direct laser writing, 3D lithography, and micro-transfer molding [160]. Furthermore, a dual-curing approach combining radical and thiol-ene photopolymerization may be employed to develop vanillin-based thermo-responsive shape memory photopolymers [158].

In another work, allyl-functionalized quercetin, a biobased polyphenolic compound, was used for thiol-ene photopolymerization, together with a biobased multifunctional thiol derived from mercaptopropionic acid and glycerol. This led to the production of thermoset material with high T_g_ and storage modulus, mainly due to the aromatic structure of the quercetin-based monomer [176].

In the composite field, by employing polymerization, copolymers of methacrylated eugenol and methacrylated coumarin derivatives were synthesized. Copolymers were then coupled with microfibrillated cellulose or hemp nanocellulose reinforcements and subsequently crosslinked, exploiting coumarin [2+2] photocycloaddition when exposed to UVA light [177,178].

The photocurable monomers obtained from different natural sources and the photoinduced polymerization processes that they can undergo, reported in detail above, are summarized in Table 2. 

## 5. Conclusions

As shown, the introduction of biobased monomers into a photopolymerizable formulation opens up new possibilities for a more environmentally friendly and greener polymer production, coupling both the sustainability of biobased precursors and energy-efficient photocuring processes. Thanks to the versatility of photocuring reactions, different network architectures can be obtained, exploiting different reactive groups and reaction pathways. Moreover, different wavelengths can be used thanks to the vast variety of available photoinitiating and photosensitizing systems. Starting from naturally derived fats and oils and waste sources, a large selection of photocurable monomers can be obtained, directly taking advantage of occurring functionalities (e.g., fatty acid unsaturations) or by straightforward modification through functional group introduction (e.g., epoxide or acrylic moieties). Even if there are still challenges to overcome, particularly in improving thermomechanical properties, e.g., by the addition of natural fillers such as cellulose, these biobased strategies offer significant opportunities in different fields of application, from the coating to the 3D printing sector.

## Figures and Tables

**Table 1:** Fatty acid composition of sunflower commercial oil (SCO) and purified WCO, determined by ^1^H NMR spectroscopy; IV = iodine number. Adapted with permission from [107], copyright Elsevier (Amsterdam, The Netherlands).

Sample	Linolenic Acid [%]	Linoleic Acid [%]	Oleic Acid [%]	SFA [%]	IV
SCO	1.9	35.7	46.4	14.0	106
WCO treated at pH = 3 and T = 25 °C	1.8	17.0	63.7	15.7	88
WCO treated at pH = 9 and T = 25 °C	1.7	17.6	63.6	15.4	89

**Table 2:** Examples of synthesized photocurable biobased monomers with relative photoinduced polymerization process.

Sources	Extracted Compound	Synthesized Monomer	Photoinduced Polymerization Process	References
Soybean	Soybean oil	Epoxidized soybean oil	Cationic	[112,113,116,129]
			FRPCP	[119,144]
		Acrylated epoxidized soybean oil	Free radical	[20,123,126,131,132,133,165]
			Thiol-ene	[120]
		Methacrylated epoxidized soybean oil	Free radical	[125]
		Cinnamate esters of epoxidized soybean oil	[2+2] photocycloaddition	[122]
Flax	Linseed oil	Linseed oil	Thiol-ene	[136,137]
		Epoxidized linseed oil	Cationic	[112,115]
			FRPCP	[119]
			Anionic thiol-epoxy	[120]
		Acrylated epoxidized linseed oil	Thiol-ene	[142]
Ricinus	Castor oil	Epoxidized castor oil	Cationic	[114,117]
		Acrylated castor oil	Free radical	[148]
			Thiol-ene	[138]
		Allylated castor oil	Thiol-ene	[138]
		Castor oil-based thiol	Thiol-ene	[140]
Cotton	Cottonseed oil	Epoxidized cottonseed oil	Anionic thiol-epoxy	[121]
Rapeseed	Rapeseed oil	Acrylated rapeseed oil	Free radical	[127]
Vernonia	Vernonia oil	Vernonia oil	Cationic	[116]
Rose	Rose hip seed oil	Epoxidized rose hip seed oil	Cationic	[118]
Grape	Grapeseed oil	Epoxidized grapeseed oil	Cationic	[118]
		Acrylated grapeseed oil	Free radical	[127]
Citrus	Limonene	Limonene	Free radical	[145]
		1,2-Limonene oxide	Cationic	[143]
			Thiol-ene	[143]
		Limonene dioxide	Cationic	[143]
			FRPCP	[144]
Cashew nut	Cardanol	Epoxidized cardanol	Cationic	[88,148,151,152,153]
		Acrylated cardanol	Free radical	[163,164,165,166,167,168,169,170]
		Methacrylated cardanol	Free radical	[150,161,162]
Cloves Nutmeg Cinnamon Lignin	Eugenol	Epoxidized eugenol	Cationic	[154]
		Eugenol diglycidyl ether	Cationic	[155]
		Acrylated eugenol	Free radical	[124,157]
		Methacrylated eugenol	Free radical	[135,171,172,173]
		Allylated eugenol	Thiol-ene	[94,99,174,175]
		Thiolated eugenol	Thiol-ene	[142]
		Eugenol and coumarin methacrylic latex	[2+2] photocycloaddition	[177,178]
Vanilla beans Lignin	Vanillin	Glycidylated vanillin	Cationic	[117]
		Acrylated vanillin	Free radical	[160]
			Thiol-Michael/free radical	[158,159]
		Methacrylated vanillin	Free radical	[157,160,172]
			Thiol-Michael/free radical	[158,159]

## Data Availability

No new data have been generated for this review.
